# Characterization and Fine Mapping of a Blast Resistant Gene *Pi-jnw1* from the *japonica* Rice Landrace Jiangnanwan

**DOI:** 10.1371/journal.pone.0169417

**Published:** 2016-12-30

**Authors:** Ruisen Wang, Nengyan Fang, Changhong Guan, Wanwan He, Yongmei Bao, Hongsheng Zhang

**Affiliations:** State Key Laboratory of Crop Genetics and Germplasm Enhancement, Jiangsu Collaborative Innovation Center for Modern Crop Production, College of Agriculture, Nanjing Agricultural University, Nanjing, China; Fujian Agriculture and Forestry University, CHINA

## Abstract

Rice blast is a destructive disease caused by *Magnaporthe oryzae*, and it has a large impact on rice production worldwide. Compared with leaf blast resistance, our understanding of panicle blast resistance is limited. The *japonica* landrace Jiangnanwan from Taihu Lake region in China shows highly resistance to panicle and leaf blast. In this study, three generations (F_2:5_, F_2:6_, F_2:7_) consisting of 221 RILs (recombination inbreeding lines), developed from the cross of Jiangnanwan and Suyunuo, a susceptible-blast *japonica* variety, were evaluated for panicle blast resistance in the fields and leaf blast resistance in greenhouse in Nanjing in 2013, 2014 and 2015. A blast resistance gene *Pi-jnw1* referring to panicle blast resistance and leaf blast resistance was identified in the three generations and located in the region of RM27273 and RM27381 in chromosome 11. The RIL18 line harboring *Pi-jnw1* was selected to be backcrossed with Suyunuo to develop BC_2_F_2_ populations. According to the genotyping of 1,150 BC_2_F_2_ individuals and panicle blast and leaf blast resistance evaluation of 47 recombinants between RM27150 and RM27381, *Pi-jnw1* was finally mapped to the 282 kb region between markers W28 and BS39. This study revealed that Jiangnanwan harboring a panicle blast and leaf blast resistance gene *Pi-jnw1* could be a genetic source for breeding new rice cultivars with panicle blast resistance.

## Introduction

Rice blast, caused by the fungus pathogen *Magnaporthe oryzae*, is one of the most destructive diseases worldwide, and it occurred in all stages of rice growth [1,2]. The disease pathosystem comprises two major interrelated phases: leaf blast and panicle blast [3]. Compared with leaf blast resistance, less is known about the genetic components for panicle blast resistance, which is indispensable for stable rice production. Leaf blast resistant cultivars may be susceptible to panicle blast, and it implies that the genetic mechanisms of blast resistance might vary across the plant growth stages [4–7]. The technical problems as lacking of standard inoculation and evaluation systems, variations in heading date and weather conditions, are obstacles to the exploration of new gene resources of rice panicle blast resistance. Up to date, only *Pb1* was cloned from the *indica* cultivar Modan conferring to the panicle blast resistance [3,8]. It encodes a NBS-LRR protein, and can protect WRKY45 from degradation by ubiquitin proteasome system. The blast resistance of cultivar usually can be lost after few years for the genetic instability and pathogenic variability of *M*. *oryzae* [9]. Therefore, to further explore new resistance genes especially panicle blast resistance genes from rice landraces will be the most useful strategy in rice blast resistance breeding.

Up to date, approximately 100 blast resistance loci or genes have been mapped on 12 chromosomes except chromosome 3 [6, 7]. Twenty five blast resistance genes have been cloned [10], and eight of them located in two gene clusters, including three genes *Pi2*, *Pi9* and *Piz-t* in *Pi2* locus and five genes *Pik*, *Pik-m*, *Pik-p*, *Pi1* and *Pi-ke* in *Pik* locus [11–17]. Among 25 cloned genes, 23 genes encode NBS-LRR (nucleotide-binding site -leucine-rich repeat) proteins, except *Pi21* encodes proline-containing protein and *Pid2* encodes receptor kinase [18–20]. It has been shown that at least six *R* genes, *Pi1*[21], *Pi2* [22], *Pi9* [23], *Pi5* [24], *Pi33* [25] and *Pigm*[26], probably confer broad-spectrum resistance to a number of isolates from different countries respectively. For instance, *Pi9* located on the same region with *Pi2*, showed resistance to 43 isolates from 13 countries [23]. *Pi5*, a locus associated with resistance to at least 6 blast races from Philippines and 26 isolates from Korea [24], and *Pi33* was resistance to more than 2,000 isolates originating from 55 countries [25].

In our previous research, Jiangnanwan, a japonica rice landrace from Taihu Lake region of China, exhibited broad-spectrum resistance to rice blast [27]. Li et al. [28] concluded that two effect genes might be involved in the leaf blast resistance with F_2_ population deriving from a across between Jiangnanwan and a blast-susceptible variety Suyunuo. In this study, we obtained 221 F_2:7_ RILs with three generations (F_2:5_, F_2:6_ and F_2:7_), and identified panicle blast and leaf blast resistance genes to the strain Hoku 1 with QTL mapping method. Furthermore, we examine the correlation between the resistance of panicle and leaf blast, and fine mapped the blast resistance gene *Pi-jnw1*.

## Materials and Methods

### Plant materials and growth

**Fig 1:**
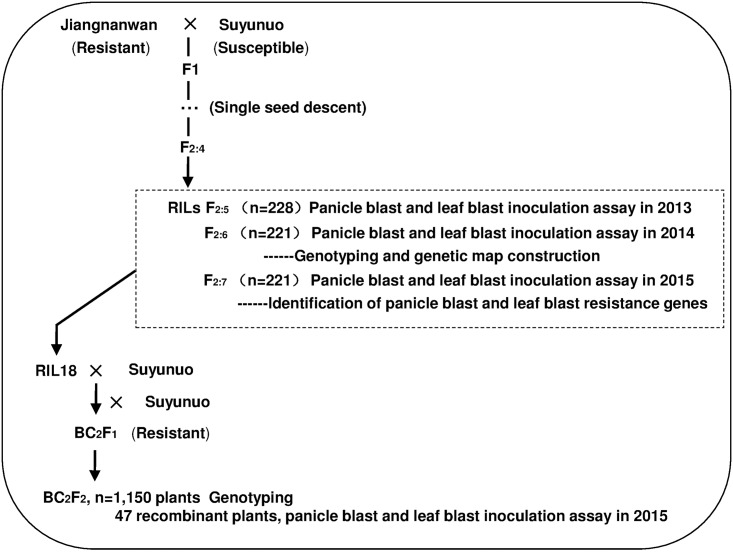
Flowchart showing the development of plant materials used in this study.

Jiangnanwan is a *japonica* rice landrace from Taihu Lake region in China and has broad spectrum resistance to leaf blast. Suyunuo is a susceptible *japonica* rice landrace from Taihu Lake region. We developed an F_2_ population from a cross between Jiangnanwan and Suyunuo, and three generations (F_2:5_, F_2:6_ and F_2:7_) of 221 recombination inbreeding lines (RILs) were generated by a single-seed descent method (Fig 1).

The populations of F_2:5_, F_2:6_, F_2:7_ and two parents (Jiangnanwan and Suyunuo) were evaluated for panicle blast resistance in 2013, 2014 and 2015 at Jiangpu Experimental Station of Nanjing Agricultural University (Jiangsu Province, China; 118°50′E, 32°02′N). Twenty plants of each RIL grew in two rows per test plot, and the spaces were 30 cm between rows and 10 cm between plants within rows. Suyunuo and Jiangnanwan were planted adjacent to the test rows as susceptible and resistant controls respectively. Field management was carried out in accordance with the local production process [29].

The populations of F_2:5_, F_2:6_, F_2:7_ and two parents (Jiangnanwan and Suyunuo) were also evaluated for leaf blast resistance in 2013, 2014 and 2015 in the greenhouse. The seeds were sown in plastic trays of 60 × 30 × 5 cm with sieved garden soil as described by Wang et al. [27]. Thirty lines and two parents were sown in each tray, and 6–8 seeds per line were sown for inoculation. Seedlings were grown in greenhouse at 22–30°C with a light and dark cycle of 16 h and 8 h until they were at the four-leaf stage for disease evaluation.

### Inoculation and disease evaluation

To evaluate the panicle blast resistance in the field, 221 RILs (F_2:5_, F_2:6_, F_2:7_), forty-seven BC_2_F_2_ recombinants and two parents at the mid-booting stage were inoculated with the strain Hoku 1 of *M*. *oryzae* by the injecting method as described as Liu et al. [30]. Fifteen booting panicles of each line were injected by 1–2 ml blast isolate Hoku 1 conidial suspension (5×10^4^ conidia/ml). Three weeks after inoculation, phenotypic evaluation was conducted based on visual assessment of diseased grains percentage as described by Koizumi et al.[31] and the scores were ranged from 0 (without diseased grain) to 100% (100% diseased grains).

Four-leaf stage rice seedlings of Jiangnanwan, Suyunuo, 221 RILs (F_2:5_, F_2:6_, F_2:7_), and forty-seven BC_2_F_2_ recombinants were inoculated with the strain Hoku1 spore suspension (5×10^4^ conidia/ml) in inoculation chambers as the method described by Wang et al. [27]. After inoculation, the plants were kept in dark at 26°C with relative humidity 95% for 24 h, and then transferred to a greenhouse with 25–28°C and 100% relative humidity by intermittently spraying water for 2 min every three hours. After seven days of inoculation, lesion scores of 0 to 5 were investigated based on lesion type with appropriate reference of the disease area of each plant as described by Shi et al.[32]. Each line was inoculated with three replications in each experiment and three independent experiments were conducted either leaf blast or panicle blast resistance evaluation.

### Genetic map construction and identification

221 RILs of F_2:6_ population were used for genotyping and constructing molecular linkage map 0.2 to 0.5 g of leaves at the four-leaf stage from each line of RILs (F_2:6_) and parents were collected specifically for DNA extraction by using the CTAB method [33]. 2,300 SSR markers kept in our lab and 108 newly designed InDel markers distributed on 12 chromosomes were screened for polymorphisms. 93 markers with polymorphisms between the two parents were finally used for genetic map construction.

All of the PCR reactions with the markers used a 10 μl reaction mixture containing of 1 μl template DNA, 0.5 μl of each primer, 0.1 μl of Taq (0.01U/μl), 1.6 μl of 10×Buffer, 0.2 μl of dNTP and 6.1 μl of ddH_2_O. PCR procedures were conducted as follows: Preheating for 5 min at 95°C, 32 cycles (40 sec at 95°C, 40 sec annealing temperature, and 40 sec at 72°C), finally 72°C for 10 min. The PCR products were analyzed on the 8% acrylamide gels.

In order to identify panicle blast and leaf blast resistance genes, QTL mapping method was performed using software IciMapping v4.0 (http://www.isbreeding.net/). The software was set LOD > 2.5 as a threshold which must be operate 1000 times at the p< 0.05 level. In this study, the panicle blast resistance QTLs were named as *qPbj-A-B*, and the leaf blast resistance QTLs were named as *qLbj-A-B*, in which A means the chromosome number and B means the sequence of QTL.

### Data analysis

Experimental data were analyzed using the IBM SPSS Statistics software 19.0, and bivariate analysis method were used for analyzing the correlation between the panicle blast resistance and leaf blast resistance [33].

### Fine mapping of *Pi-jnw1*

All of the simple sequence repeat (SSR) markers in this study were downloaded from the gramene database (http://www.gramene.org/) [34]. Five InDel markers W26, W28, BS33, BS39 and BS71 were designed on the basis of sequence difference between 93–11 (http://www.genomics.org.cn/) and Nipponbare (http://www.ncbi.nlm.nih.gov/) in the target region by Pairwise BLAST (http://blast.ncbi.nlm.nih.gov/blast.cgi) as described as Wu et al. [35] (S1 table).

The RIL18 line harboring *Pi-jnw1* was selected from F_2:6_ RIL populations and backcrossed with Suyunuo for developing fine mapping populations,.1,150 plants of BC_2_F_2_ population generated by 26 resistant BC_2_F_1_ individuals were used for constructing fine genetic linkage map and identifying recombinants in the target region of *Pi-jnw1*. The franking markers RM27150 and RM27381 were used to genotype the 1,150 BC_2_F_2_ segregating plants and 47 recombinants were detected. Then the 47 recombinants were inoculated to identify the panicle blast resistance phenotypes and the next generation seeds of 47 recombinants were inoculated to identify the leaf blast resistance phenotypes.

## Results

### Characterization of resistance to panicle and leaf blast in Jiangnanwan

**Fig 2:**
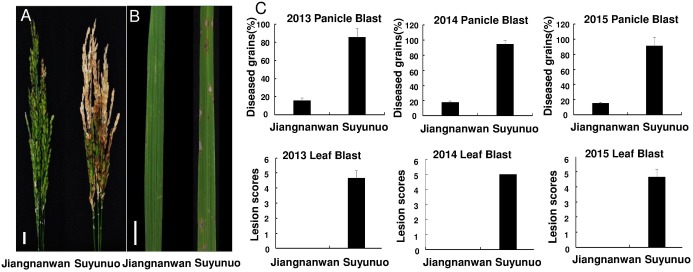
The resistant phenotypes of Jiangnanwan and Suyunuo at seedling and heading stage after inoculating by Hoku 1. A, The phenotypes of panicle blast in Jiangnanwan and Suyunuo. Bar = 1cm. B, The phenotypes of leaf blast in Jiangnanwan and Suyunuo. Bar = 1 cm. C, Characterization of panicle and leaf blast severity distribution of Jiangnanwan and Suyunuo in three years.

**Fig 3:**
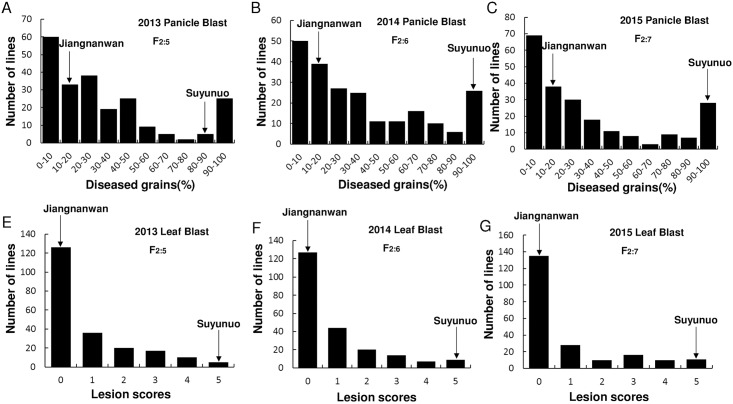
Characterization of panicle and leaf blast severity distribution in RILs F_2:5_, F_2:6_, and F_2:7_.

In 2013, 2014 and 2015, Jiangnanwan and Suyunuo were inoculated with the strain Hoku1 in field for panicle blast resistance evaluation and in greenhouse for leaf blast resistance evaluation. The results showed that Jiangnanwan was highly resistance to panicle blast and leaf blast, while Suyunuo was highly susceptible (Fig 2A–2C, Table 1). The frequency distributions of panicle blast and leaf blast resistance in 221 RILs (F_2:6_, F_2:7_ and F_2:8_) were asymmetric and continuous, and they were all predisposed resistance-inclined distribution (Fig 3A–3G). Similar results of frequency distributions of panicle blast and leaf blast resistance were obtained by the IBM SPSS Statistics software 19.0, and the characteristic parameters (Skewness and Kurtosis) showed the frequency distributions of panicle blast and leaf blast resistance in 221 RILs were all predisposed resistance-inclined distribution (Table 1).

**Table 1:** Phenotypic values of panicle and leaf blast resistance to strain of Hoku 1 in RILs F_2:5_, F_2:6_, and F_2:7_ populations.

Blast resistance type	Year	Parents		RIL Population ^c^					
		Jiangnanwan	Suyunuo	Mean	Max	Min	SD ^d^	Skewness	Kurtosis
Panicle blast resistance ^a^	2013	15.97±2.64% (R)	85.67±9.56% (S)	39.66%	100%	0	0.32371	0.888	-0.563
	2014	18.2±3.45%(R)	100% (S)	38.04%	100%	0	0.31704	0.736	-0.735
	2015	15.46±1.21%(R)	91.67±11.11% (S)	33.09%	100%	0	0.32573	1.011	-0.333
Leaf blast resistance ^b^	2013	0(R)	5(S)	1	5	0	1.29192	1.459	1.199
	2014	0(R)	5(S)	1	5	0	1.43337	1.486	1.27
	2015	0(R)	5(S)	1	5	0	0.64204	2.329	5.321

^a^ means diseased grains (%);

^b^ means lesion score;

^c^ RILs sample size n = 221, replications r = 3;

^d^ standard deviation.

The frequency distributions of panicle blast resistance in three generations of 221 RILs (F_2:5_, F_2:6_ and F_2:7_) were asymmetric and continuous, and they were all predisposed resistance-inclined distribution (Fig 2A–2C). The frequency distributions in the three tested generations under the experimental paddy field and greenhouse were not bimodal, suggesting that multiple loci are involved in the panicle blast and leaf blast resistance of Jiangnanwan (Fig 2D–2F).

### Identification of *Pi-jnw1*

**Fig 4:**
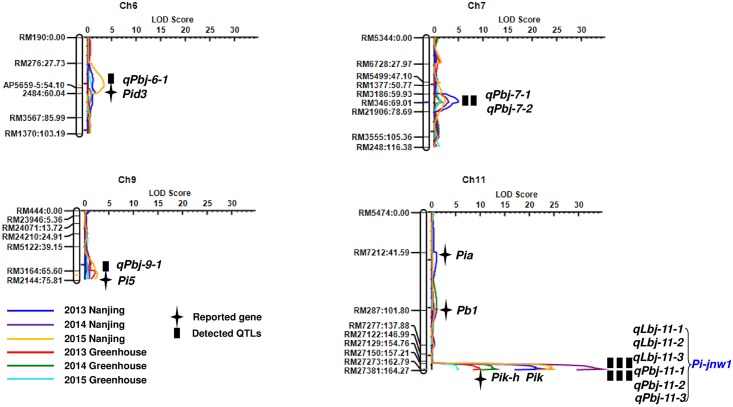
Identification of panicle and leaf blast resistance genes in Jiangnanwan by QTL mapping method. Marker names and their positions were showed on the left linkage group. The color lines indicated LOD scores.

With a linkage map which covering 1,690.76 cM on the 12 chromosomes and an average distance 18.18 cM between two connected markers, a blast resistance gene *Pi-jnw1* referring to panicle blast and leaf blast resistance was detected in the same region of RM27273 and RM27381 on chromosome 11 by QTL IciMapping 4.0 in three generations (F_2:5_, F_2:6_ and F_2:7_) (Table 2, Fig 4).

**Table 2:** Identification of panicle and leaf blast resistance genes by QTL mapping method in RILs F_2:5_, F_2:6_, and F_2:7_ populations.

Traits	Generation	QTL Names	Chr.	Left Marker	Right Marker	LOD	PVE (%)	Add
Panicle blast	F2:5	*qPbj-7-1*	7	RM3186	RM346	4.8629	6.9138	0.0849
		*qPbj-11-1 (Pi-jnw1)*	11	RM27273	RM27381	23.2412	39.9217	-0.2045
	F2:6	*qPbj-7-2*	7	RM3186	RM346	2.8482	2.8882	0.0536
		*qPbj-11-2 (Pi-jnw1)*	11	RM27273	RM27381	34.7518	53.6856	-0.2321
	F2:7	*qPbj-6-1*	6	RM276	AP5659-5	3.2884	5.2431	0.0766
		*qPbj-9-1*	9	RM3164	RM2144	2.5526	3.9206	-0.0644
		*qPbj-11-3 (Pi-jnw1)*	11	RM27273	RM27381	24.7161	42.4911	-0.2130
Leaf blast	F2:5	*qLbj-11-1 (Pi-jnw1)*	11	RM27273	RM27381	9.7449	18.8430	-0.5623
	F2:6	*qLbj-11-2 (Pi-jnw1)*	11	RM27273	RM27381	13.1284	23.6070	-0.6635
	F2:7	*qLbj-11-3 (Pi-jnw1)*	11	RM27273	RM27381	5.3520	10.9116	-0.3286

For the panicle blast resistance, *Pi-jnw1* was detected in the same region of RM27273 and RM27381 on chromosome 11 (*qPbj-11-1*, *qPbj-11-2* and *qPbj-11-3*) in 2013, 2014 and 2015, respectively (Fig 4). They could explain 39.92%, 53.68% and 42.49% of phenotypic variation for panicle blast resistance in three generations (Table 2). Other four minor resistant loci, including *qPbj-7-1*, *qPbj-7-2*, *qPbj-6-1* and *qPbj-9-1* detected in 2013, 2014, and 2015, respectively could explain only 2.89%-6.91% of phenotypic variation (Table 2). Among these four minor resistant loci, *qPbj-7-1* and *qPbj-7-2* were located in the same region of RM3186 and RM346 on chromosome 7, and *qPbj-6-1* and *qPbj-9-1* were located in the region of RM276 and AP5659.5 on chromosome 6 and in the region of RM3164 and RM2144 on chromosome 9, respectively (Fig 4).

For the leaf blast resistance, *Pi-jnw1* was also detected in the same region of RM27273 and RM27381 on chromosome 11 (*qLbj-11-1*, *qLbj-11-2* and *qLbj-11-3*) in 2013, 2014 and 2015, respectively (Fig 4). They could explain 18.84%, 23.60%, and 10.91% phenotypic variation for leaf blast resistance in three generations.

To determine whether leaf blast resistance was related with panicle blast resistance in Jiangnanwan, the correlation of panicle blast resistance and leaf blast resistance of 221 F_2:5_, F_2:6_, F_2:7_ RILs was examined. The results showed that the correlation coefficients between panicle blast resistance and leaf blast resistance were 0.49 in the F_2:5_ RILs, 0.371 in the F_2:6_ RILs, and 0.551 in the F_2:7_ RILs respectively, all with a significantly positive relationship (Table 3).

**Table 3:** The correlations between panicle and leaf blast resistance reaction.

		**Panicle blast**		
	**Generation**	**F_2:5_**	**F_2:6_**	**F_2:7_**
**Leaf blast**	F2:5	0.49**		
	F2:6	—	0.371*	
	F2:7	—	—	0.551**

“**” P<0.01,

“*” P<0.05.

### Fine mapping of *Pi-jnw1*

**Fig 5:**
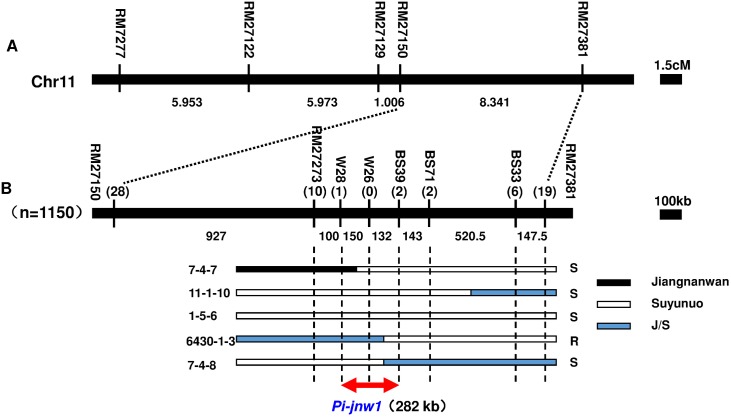
Fine mapping of *Pi-jnw1*. A. A total of 47 recombinant plants were screened from 1150 BC_2_F_2_ segregating plants. B. High resolution analysis of phenotypes and genotypes. Serial numbers represent partial key recombinant plants. The black regions indicated Jiangnanwan genotypes, the white regions indicated Suyunuo genotypes and the blue regions indicated hybrid subtype of Jiangnanwan/Suyunuo.

Five InDel markers W26, W28, BS33, BS39 and BS71 between RM27273 and RM27381 with polymorphisms between two parents Jiangnanwan and Suyunuo were used to fine map the *Pi-jnw1*. 1,150 BC_2_F_2_ plants were genotyped by those markers, and 28, 10, 1, 0, 2, 2, 6 and 19 recombinants were identified by RM27150, RM27273, W28, W26, BS39, BS71, BS33 and RM27381, respectively. Through panicle blast and leaf blast resistance phenotype assays of the forty seven recombinants between RM27150 and RM27381, *Pi-jnw1* was mapped in the region of W28 and BS39 with the physical distance of 282 Kb (Fig 5).

## Discussion

Panicle blast usually caused more loss of yielding than leaf blast in rice production. However, fewer genetic analyses of rice panicle blast resistance have been reported compared with leaf blast resistance. More field works, complex phenotype evaluation system and the influence of environmental conditions are obstacles to study rice panicle blast resistance. There are various ways for evaluating the panicle blast resistance: (1) Injecting inoculation, injecting 2–3 ml of spore suspension into one booting panicle [30]; (2) Inducing inoculation, controlling the field conditions to be suitable for development and epidemic of blast disease [3]; (3) *In vitro* inoculation, 6 cm rice panicle necks containing 1–3 rachis nodes were put on the filter paper in petri dishes, then the nodes were inoculated with 2 ml spore suspension by a micropipette [5]. The resistance genes *Pi-64* and *Pi-25* were identified by *vitro* inoculation [35,36], and *Pb1* was identified under suitable field conditions for blast disease development [3]. We use the modified injecting method to inoculate the 221 RILs and forty-seven BC_2_F_2_ recombinants in the field. In our study, the frequency distributions of diseased grains percentages in the three tested generations in different fields were relatively stable and a blast resistance gene *Pi-jnw1* referring to panicle blast resistance in chromosome 11 were detected in three years and two minor resistant loci (*qPbj-7-1* and *qPbj-7-2*) in chromosome 7 were both detected in two years. It indicated that the injecting inoculation method might be more appropriate for identifying the panicle blast resistance.

Jiangnanwan, one *japonica* rice landrace from Taihu Lake region in China, exhibited broad-spectrum resistance to leaf blast and highly resistance to panicle blast [37]. Li et al. [28] studied the genetic patterns of leaf blast resistance to *Hoku1* in Jiangnanwan with P_1_, P_2_, F_1_ and F_2_ population deriving from a across between Jiangnanwan and a blast-susceptible variety Suyunuo and concluded that two genes might be involved. In our results, only one blast resistance gene *Pi-jnw1* could be detected in 221 F_2:5_, F_2:6_, F_2:7_ RILs, respectively. The possible reason could be due to the different populations and different analysis methods.

So far, more than 20 blast resistance genes were reported on rice chromosome 11, four of them locate near *Pi-jnw1* region. The *Pb1* locus was mapped in the Modan-derived chromosomal region in the middle part of the long arm of chromosome 11, located closet with the RFLP marker of S723[3]. The rice blast resistance gene *Pik*, which confers high and stable resistance to many Chinese rice blast isolates, encoded two coiled-coil nucleotide binding site leucine-rich repeat (NBS-LRR) proteins[17]. The *Pi34* locus was located in the 54.1 kb region on the genomic sequence of Nipponbare and acted partial resistance to blast in Chubu 32[38]. *Pi-hk1* was identified on chromosome 11 of Heikezijing, located between the SSR markers of RM7654 and RM27381[20]. According to the fine mapping results, *Pi-jnw1* was not in the same region of *Pb1*. In our further study, we will confirm the fine mapping results and use more markers to detect whether Jiangnanwan harbors *Pik*, *Pi34* and *Pi-hk1* genes in the *Pi-jnw1* region.

In our study, the blast resistance gene *Pi-jnw1* was identified both in panicle blast resistance and leaf blast resistance of the three generations (F_2:5_, F_2:6_ and F_2:7_), suggesting that there was a positive relationship between panicle blast and leaf blast resistance detected in Jiangnanwan. It is consistent with the common viewpoint that panicle blast resistance is correlated with leaf blast resistance in many rice cultivars[39]. However, there were also four minor panicle blast resistance specific loci *qPbj-6-1*, *qPbj-7-1*, *qPbj-7-2* and *qPbj-9-1*, and it indicates that some loci might be only influence the panicle blast resistance. Interestingly, *qPbj-7-1*, *qPbj-7-2* and *qPbj-6-1* were contributed by Suyunuo indicated that there were some genes in Suyunuo against panicle blast which could be detected under specific conditions. In this study, 93 genetic markers with polymorphisms between two parents were used for genetic map construction, and the frequency of polymorphisms between Jiangnanwan and Suyunuo was not as high as the *indica*/*japonica* crosses. In further research, more genetic markers between W28 and BS39 will be designed and larger segregation populations will be constructed for fine mapping the *Pi-jnw1*. The recombinants harboring *Pi-jnw1* will be further used for breeding new cultivars with back crossing with the elite cultivars and marker associated selection method (MAS).

Breeding new blast resistant cultivars is considered as an effective and economical way to control this disease. However, among the cloned 25 resistance genes, 24 of them were referring to the leaf blast resistance and few of them have been widely applied in rice breeding. Many cultivars show different levels of partial resistance to leaf and panicle blast. This implies that the genetic mechanisms of host resistance might vary across growth stages. In this study, Jiangnanwan showed broad spectrum resistance to leaf blast and highly resistance to panicle blast, and the panicle blast resistance showed a positive correlation with leaf blast resistance. The mapping results also showed that *Pi-jnw1* could be detected with panicle blast resistance phenotypic data and leaf blast resistance phenotypic data in three years and located in the same region of RM27273 and RM27381 on chromosome 11 in the three generations (F_2:5_, F_2:6_ and F_2:7_). It indicated that Jiangnanwan might be a good resource for application in rice breeding programs, and further cloning and functional analysis of *Pi-jnw1* could be necessary for clarifying the molecular basis of panicle blast resistance and leaf blast.

## Supporting Information

S1 Table Information on polymerase chain reaction (PCR)-based markers used for mapping of *Pi-jnw1*. (DOCX)
