# Supplementary material for: Characterization and Fine Mapping of a Blast Resistant Gene Pi-jnw1 from the japonica Rice Landrace Jiangnanwan
Source: PLoS One. 2016 Dec 30;11(12):e0169417. doi: 10.1371/journal.pone.0169417 (PMC5201426; doi:10.1371/journal.pone.0169417)
Supplement: S1 Table — (DOCX) [file pone.0169417.s001.docx]

**S1 table** Information on polymerase chain reaction (PCR)-based markers used for mapping of *Pi-jnw1*.

| **Marker** | **Chromosome** | **Primer sequence (5′–3′)** | **Anneal temperature (°C)** | **Product size (bp)** | **Position** |
| --- | --- | --- | --- | --- | --- |
| W26 | 11 | F:CCCTTTAACACATTCCAGCAA | 55 | 177 | 29447611 |
|  |  | R:CCCACCCCAATCTAGCAAT |  |  |  |
| W28 | 11 | F:ATCTGTTACTGTTAGACGGC | 55 | 201 | 29330882 |
|  |  | R:GTACTACTTCACATGAGGAA |  |  |  |
| BS33 | 11 | F:CTGATCTCAATCAGACAGAA | 55 | 173 | 30065736 |
|  |  | R:GCTATCTTTGCCGAATGAG |  |  |  |
| BS39 | 11 | F:CTTCTGGATCTCTTCACTGA | 55 | 131 | 29534270 |
|  |  | R:TTGAGCATGACATTGAGGA |  |  |  |
| BS71 | 11 | F:TGTCTATTGCGTTGATTTG | 55 | 186 | 29677709 |
|  |  | R:CCGCCAGCTTGCTCTT |  |  |  |
